# Protection of xenon against postoperative oxygen impairment in adults undergoing Stanford Type-A acute aortic dissection surgery: Study protocol for a prospective, randomized controlled clinical trial

**DOI:** 10.1097/MD.0000000000007857

**Published:** 2017-08-25

**Authors:** Mu Jin, Yi Cheng, Yanwei Yang, Xudong Pan, Jiakai Lu, Weiping Cheng

**Affiliations:** a Department of Anesthesiology; b Department of Cardiology, Beijing Anzhen Hospital, Capital Medical University, Beijing Institute of Heart Lung and Blood Vessel Diseases, Beijing, China.

**Keywords:** acute aortic dissection surgery, oxygen impairment, postoperative, protocol, xenon

## Abstract

**Objectives:**

The available evidence shows that hypoxemia after Stanford Type-A acute aortic dissection (AAD) surgery is a frequent cause of several adverse consequences. The pathogenesis of postoperative hypoxemia after AAD surgery is complex, and ischemia/reperfusion and inflammation are likely to be underlying risk factors. Xenon, recognized as an ideal anesthetic and anti-inflammatory treatment, might be a possible treatment for these adverse effects.

**Methods/Design:**

The trial is a prospective, double-blind, 4-group, parallel, randomized controlled, a signal-center clinical trial. We will recruit 160 adult patients undergoing Stanford type-A AAD surgery. Patients will be allocated a study number and will be randomized on a 1:1:1:1 basis to receive 1 of the 3 treatment options (pulmonary inflated with 50% xenon, 75% xenon, or 100% xenon) or no treatment (control group, pulmonary inflated with 50% nitrogen). The aims of this study are to clarify the lung protection capability of xenon and its possible mechanisms in patients undergoing the Stanford type-A AAD surgery.

**Discussion:**

This trial uses an innovative design to account for the xenon effects of postoperative oxygen impairment, and it also delineates the mechanism for any benefit from xenon. The investigational xenon group is considered a treatment intervention, as it includes 3 groups of pulmonary static inflation with 50%, 75%, and 100% xenon. It is suggested that future trials might define an appropriate concentration of xenon for the best practice intervention.

**KEY POINTS**

- This is the first randomized-controlled trial assessing xenon protection against postoperative oxygen impairment in adults undergoing Stanford Type-A acute aortic dissection surgery.
- This study will determine the potential of different concentration of xenon-treatment against postoperative oxygen impairment in adults undergoing Stanford Type-A acute aortic dissection surgery.
- This study will determine pathophysiological mechanisms of xenon-treatment on postoperative acute oxygen impairment.
- This study will conducted in the largest aortic medicine clinic center of China and large nationally representative sample of the general population in China was collected. However, this is a single-center study; therefore, the applicability of the results to other medical center would be affected and a multicenter large cohort study is recommended.

## 1 Introduction

Acute aortic dissection (AAD) is a severe cardiovascular disease demonstrating the characteristics of acute onset and rapid development, with high morbidity and mortality.^[1]^ Previous work by Wang et al^[2]^ showed that the incidence of postoperative hypoxemia is up to 49.5% in adult patients undergoing Stanford A AAD surgery. Postoperative hypoxemia was always related with postoperative acute lung injury (ALI), even acute respiratory distress syndrome (ARDS) according to the “Berlin criteria” of 2012 by the European Society of Intensive Care Medicine.^[3]^ The pathogenesis of hypoxemia after AAD surgery is complex and remains unknown. However, studies have indicated that ischemia/reperfusion and inflammation are likely to be underlying risk factors.^[4–6]^ Therefore, reducing inflammation might be a potential therapeutic strategy for treating postoperative hypoxemia.

Xenon has been recognized as an ideal anesthetic for its hemodynamic stability and anti-inflammatory effects.^[7–9]^ In recent years, it has also been demonstrated to have neuroprotective and cardioprotective effects by inhibiting inflammation in different models subjected to preconditioning, real-time conditioning, and postconditioning.^[10–13]^ However, the effects of xenon on respiratory function in patients with Stanford type A AAD during cardiopulmonary bypass (CPB) with deep hypothermic circulatory arrest (DHCA) remain unclear. In addition, no RCTs have been performed on patients with postoperative oxygen impairment to show that xenon treatment is more effective than inhaled oxygen.

## 2 Methods/design

### 2.1 Ethical approval

Ethical approval was obtained on March 10, 2016, from the Beijing Anzhen Hospital Clinical Research Ethics Committee (2016006). The study has been accepted on to the Beijing municipal science and technology plan project. Personal information about potential and enrolled participants will be collected, shared, and maintained by sponsor in order to protect confidentiality before, during, and after the trial.

### 2.2 Design of trial

The trial is a 4-group, parallel, RCT, which has been registered at www.clinicaltrials.gov (Identifier: NCT02468531). By performing 4 2-way comparisons, we will test differences between groups. Patients will be randomized in a 1:1:1:1 ratio. Blinding will be performed for patients, post intervention assessors, statisticians, and investigators.

The null hypothesis for this trial is that there is no difference in protection effects between nitrogen and xenon in patients with postoperative oxygen impairment. The patients who receive nitrogen are the control group in this comparison. If this null hypothesis is rejected, then inhaled xenon may be an effective treatment. If this null hypothesis is not rejected, then inhaled xenon may be ineffective.

However, there is also a need to understand how potential treatment differences are achieved, and/or if different concentrations of inhaled xenon produce similar effects. Further comparisons, including the use of a control intervention, can provide this information.

### 2.3 Aims

The aims of this study are to clarify the lung protection effects of xenon and its possible mechanisms in patients undergoing the Stanford type-A AAD surgery. It includes a detailed statistical analysis of perioperative clinical baselines and serum variables, such as coagulation, fibrinolysis, inflammatory, reactive oxygen species, and endothelial cell function. The outcomes of this study will detail the differences in postoperative oxygen impairment between the control group and xenon groups, and pathophysiological mechanisms of xenon, which will be beneficial for the prophylaxis, and/or treatment of postoperative acute lung injury.

### 2.4 Primary and secondary outcome measures

#### 2.4.1 Primary outcome measure

The study primary outcome measure is the perioperative change of the patient's oxygenation index (OI) presurgery, at end of surgery, and at 6 hours postsurgery. The oxygenation index is calculated by the arterial oxygen tension (PaO_2_)/inspired oxygen fraction (FiO_2_) ratio, and is a validated and effective measure of oxygen impairment change.

#### 2.4.2 Secondary outcome measures

The following are the secondary outcome measures: inflammatory cytokines and reactive oxygen species (ROS) presurgery, at end of surgery, at 6 and 24 hours postsurgery; extubation time; and complications. The timing of all assessments is shown in Table 1 as a schedule of events.

**Table 1:**
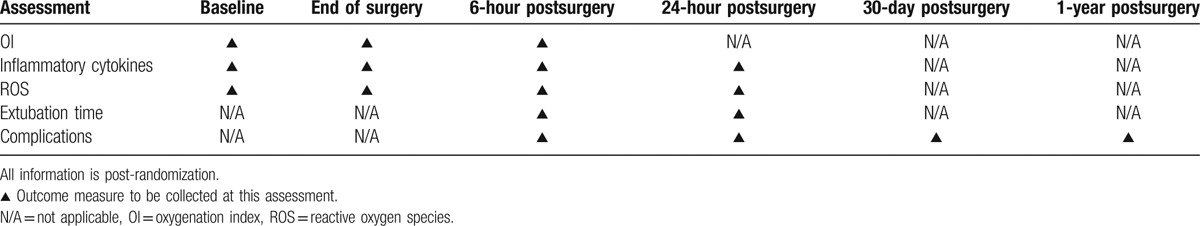
Timetable of assessments.

### 2.5 Study participants

The diagnosis of AAD will be confirmed in all patients by a consultant surgeon. A clinical history and clinical examination will be completed by recruiting surgeons on the team. Dissection is considered AAD if the time from the onset of the symptoms to surgery is 14 days or less. This study will include only type A AAD, as according to the Stanford classification.^[14,15]^

#### 2.5.1 Inclusion criteria

Patients diagnosed with Stanford type A AAD by consultant's clinical judgment using local methods of diagnosis, which include clinical history, chest radiography (x-rays), transthoracic ultrasound, and contrast-enhanced computed tomography (CT), or magnetic resonance imaging (MRI) will be included. Patients aged 18 to 65 years old, and patients eligible for AAD surgery will be included, as well.

#### 2.5.2 Exclusion criteria

A participant will not be enrolled into the study if any of the following conditions apply: coronary heart disease, heart failure, severe cardiac tamponade, unstable hemodynamics, severe nervous system abnormalities, clinically apparent malperfusion including lower limb, cerebral, coronary and renal malperfusion, and visceral ischemia or severe hepatic and renal abnormalities. Patients will also be excluded if they have undergone any cardiac and thoracic surgeries, are unlikely to be able to perform the required clinical assessment tasks, have significant cognitive impairment or language issues, are unable to provide consent to participate in the study or have been prescribed non-steroidal anti-inflammatory drugs or corticosteroids before or after hospital admission

### 2.6 Study procedures

#### 2.6.1 Recruitment organization

Patients considered for AAD will be identified in the emergency department. In all cases, the consultant cardiac surgeon will have final approval of a patient's eligibility for the study. Patients will be provided with written information about the study and asked to opt in. If their interest continues, they will be provided with further written information and evaluated for assessment and consent.

#### 2.6.2 Randomization

After written informed consent is obtained and patient eligibility confirmed, patients will have a baseline assessment. They will then be randomized into the study. Patients will be given a study number and randomized on a 1:1:1:1 basis to receive 1 of the 3 treatment options or no treatment (control group). Randomization will be performed using an automated, computer-generated randomized digital table to ensure that all 4 groups are well balanced for the patient characteristics.

#### 2.6.3 Surgery

As a previous study described^[16]^ in brief, a stent graft and a 4-branch prosthetic graft will be used for arch replacement. Valve-sparing root resection (aortic valve plasty, the David procedure, or valsalva sinus plasty) or valve graft replacement will be performed according to the subtype of the aortic root.

#### 2.6.4 Study assessments

Patients will complete their baseline assessments before surgery, and the remaining assessments are planned for the end of surgery, 6 and 24 hours postsurgery and 30-day postsurgery. Follow-up measurements will be conducted 3, 6, and 12 months after surgery.

### 2.7 Study interventions

Patients randomized to surgery will remain unaware of which procedure they underwent (xenon or nitrogen). The study surgical procedures mirror each other, with standardized anesthesia, postoperative care, and rehabilitation for both groups. The patients will be mechanically ventilated with 50% to 100% oxygen and a 5-cm H_2_O positive end-expiratory pressure (PEEP). The end-tidal carbon dioxide pressure (P_ET_CO_2_) will be maintained at between 35 and 45 mm Hg by adjusting the tidal volume to 6 to 8 mL/kg with a respiratory rate of 10 to 15 times/min. Further details of the individual interventions are listed below.

### 2.8 Nitrogen group (control group)

The nitrogen arm is the surgical comparison group. The procedure will be performed while the patient is under general anesthesia. After routine intubation, 50% nitrogen/50% oxygen will be inhaled for 1 hour, and then 100% oxygen continued until cardiopulmonary bypass (CPB) is performed. After the aorta is cross-clamped and mechanical ventilation stopped, the lungs will be inflated with 50% nitrogen/50% oxygen to maintain a mean airway pressure. Pulmonary static inflation will be stopped 15 minutes before aortic declamping and cardiac resuscitation. One hour after the end of CPB, 50% nitrogen/50% oxygen will be inhaled for 1 hour, and then 100% oxygen will be continued until the end of surgery. These measures provide the oxygen group with the characteristics necessary to provide a reasonable comparison and to account for the placebo effects of surgery.

### 2.9 Xenon group

The procedure will be performed with the patient under general anesthesia. After routine intubation, 50% xenon/50% oxygen will be inhaled for 1 hour, and then 100% oxygen continued until CPB is performed. One hour after the end of CPB, 50% xenon/50% oxygen will be inhaled for 1 hour, and then 100% oxygen will be continued until the end of surgery.

#### 2.9.1 50% xenon group:

After the aorta is cross-clamped and mechanical ventilation is stopped, the lungs will be inflated with 50% xenon/50% oxygen to maintain a mean airway pressure. Pulmonary static inflation will be stopped 15 minutes before aortic declamping and cardiac resuscitation.

#### 2.9.2 75% xenon group:

After the aorta is cross-clamped and mechanical ventilation stopped, the lungs will be inflated with 75% xenon/25% oxygen to maintain a mean airway pressure. Pulmonary static inflation will be stopped 15 minutes before aortic declamping and cardiac resuscitation.

#### 2.9.3 100% xenon group:

After the aorta is cross-clamped and mechanical ventilation stopped, the lungs will be inflated with 100% xenon to maintain a mean airway pressure. Pulmonary static inflation will be stopped 15 minutes before aortic declamping and cardiac resuscitation.

The OI will be recorded before the operation, at end of surgery, and 6 hours postoperatively. OI will be calculated based on an FiO_2_ of 100%, a PEEP of 5 cm H_2_O, a tidal volume of 8 mL/kg, an inspiratory/expiratory ratio of 1:2, and a respiratory rate of 10 times per minute.

## 3 Safety

Data on complications will be recorded as outcomes, and the severity and frequency of all complications will be assessed and reported.

### 3.1 Definition of serious adverse events

A serious adverse event (SAE) is any negative medical occurrence, including death, life-threatening injury, an change requiring inpatient hospitalization or prolongation of existing hospitalization or a change that results in persistent or significant disability and/or incapacity.

### 3.2 Reporting procedures for unexpected serious adverse events

All unexpected serious adverse event (USAE), related to any of the research procedures or not listed in the protocol, will be reported to the Beijing Anzhen Hospital Clinical Research Ethics Committee that will issue a favorable opinion of the study and compensate to those who suffer harm from trial participation. Reports of related SAEs and USAEs should be submitted within 24 hours of the principal investigators awareness of the event.

### 3.3 Follow-up and end of study definition

After 3, 6, or 12 months, the participant will be consulted in out-patient department or contacted by telephone for assessment of individual survival, living condition, and major adverse events. The whole duration of the study will be 1 year and will end after the 1-year follow-up of the last patients. Participants have no obligation to complete the whole study and they are free to decide to withdraw at any point.

### 3.4 Statistics and analysis

#### 3.4.1 Target sample size

A total sample size of 160 patients is planned. For a balanced 4-group trial, approximately 40 patients must be recruited per allocation group (nitrogen group, 50% xenon group, 75% xenon group, and 100% xenon group).

#### 3.4.2 Sample size calculation

The sample size calculation is based on the primary outcome measure, which is the variable of OI at perioperative period. Currently, there is no established minimal clinically important difference (MCID) for the OI. Based on previous study,^[17]^ an approximation to the MCID could be viewed as 38 mm Hg, the standard deviation (SD) of a single OI observation as 70 mm Hg, and the auto-correlation as 0.5. With a 5% (2-sided) type I error rate, based on preliminary experiments, 36 patients per treatment group are necessary, and the power was 0.80048. Assuming a 10% dropout rate, the final sample size will be 160 patients (40 patients/group) (Table 2). (Power Analysis and Sample Size, PASS V11.0, NCSS, LLC; Kaysville, UT).

**Table 2:**
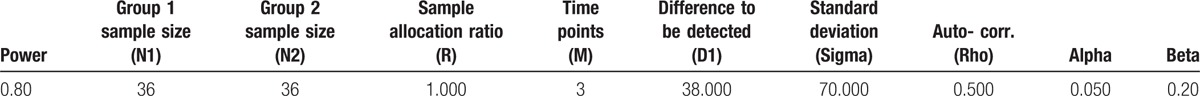
Power of calculated sample size.

#### 3.4.3 Analysis of endpoints

The statistical analysis of the study is the responsibility of the trial statistician. Baseline data will be compared to determine whether or not there are any clinically important differences between treatment and control groups that have occurred by chance. The analysis will be performed by SPSS statistical software (SPSS for Windows, V.18.0; Chicago, IL).

All statistical testing will be performed at the 2-sided 5% significance level, and 95% confidence intervals will be presented where appropriate. For all analyses, the appropriate model assumptions were verified, and alternative methods used if more appropriate. Prior to any analysis, any missing data patterns will be investigated, the reasons for missing data obtained and summarized where possible. The primary analysis, such as OI, inflammatory cytokines, and ROS, will be conducted on the repetitive measurement and analysis of variance. The intubation time will be summarized descriptively using median, interquartile ranges and ranges overall, as well as by treatment arm. Complications between the trial arms will be compared in terms of frequency and severity. Cross-tabulations will be generated to relate complications to treatment, and chi-squared tests will be used to assess the significance of the association. A receiver operating characteristic (ROC) curve will be used to test discrimination of the treatment model. A *P* value <.05 will be considered statistically significant.

#### 3.4.4 Serum sample study

Serum samples from patients in all groups will be drawn from a central vein catheter. Our aim is to analyze this tissue to investigate the relationship between variations in interleukin-6 (IL-6), interleukin-10 (IL-10), tumor necrosis factor a (TNFa), methane dicarboxylic aldehyde (MDA), myeloperoxidase (MPO), total antioxidation capacity (TAOC), total superoxide (TSOD), and clinical presentation of perioperative levels and levels with xenon/oxygen treatment. A better understanding of the key molecular processes and xenon treatment also has the potential of leading to novel treatment therapies.

## 4 Discussion

This trial protocol has several aspects that warrant further discussion, as it has an innovative design, and its main concern is xenon's potential organ protection, as outlined in the results of experiments in animals and limited studies in humans.

### 4.1 Intervention content

The intervention content for each of the groups was carefully selected. The content of the oxygen group was chosen to represent current practice for this operation. Based on a previous non-randomized controlled clinical trial,^[17]^ pulmonary static inflation with 50% xenon during CPB attenuated OI decreases at the end of surgery; but the effectiveness diminished at 6 hours after surgery and the valid intra-alveolar xenon concentration was only about 25% to 30% of the inhaled 50% xenon concentration. Therefore, a xenon-treatment arm was designed as 3 groups of pulmonary static inflation with 50%, 75%, and 100% xenon, respectively. We suggest that future trials might define appropriate concentration of xenon for the best practice intervention.

### 4.2 Compliance and loss to follow-up

There are approximately 300 to 400 cases of AAD surgery performed in the Beijing Anzhen Hospital per year. If 50% of these patients are eligible and consent, we will meet our recruitment target in approximately 1 year. Therefore, we have accounted for a dropout rate of 10% to 15% from the entire study over time. These estimates are based on assessments of our previous study data.^[16]^

### 4.3 Interpretation

The importance of highlighting potential outcomes and clinical implications has been shown by the development of this trial. If each concentration of xenon-treatment is shown to be no more effective than nitrogen-control, then the continued use of xenon for this condition and in a wider context should be terminated. Conversely, if 50% xenon (or 75% xenon or 100% xenon) is shown to be an effective treatment, then the current xenon-treatment for the procedure should be encouraged. If 100% xenon is more effective than 50% xenon, then it will have been confirmed that the effectiveness of xenon is based on concentration. If a comparison of 100% xenon with 50% xenon demonstrates no difference in primary outcome, then there are health care economic implications, and a cost utility analysis will be performed for future procedures.

### 4.4 Dissemination

Data collection will be completed by mid- or late-2018. Data analysis will start immediately after data monitoring completed. Publications will be prepared for submission in mid-2019. The results of the trial will be published according to the CONSORT statement. Dissemination of results will focus on publications in peer-reviewed journals, and presentations at national/international cardiology surgery or anesthesiology meetings to have widespread dissemination.

## Acknowledgments

The authors thank Accdon for its linguistic assistance during the preparation of this manuscript.
